# Distal femoral condyle is more internally rotated to the patellar tendon at 90° of flexion in normal knees

**DOI:** 10.1186/s13018-015-0197-5

**Published:** 2015-04-25

**Authors:** Shinya Kawahara, Ken Okazaki, Shuichi Matsuda, Hiroyuki Nakahara, Shigetoshi Okamoto, Yukihide Iwamoto

**Affiliations:** Department of Orthopaedic Surgery, Graduate School of Medical Sciences, Kyushu University, 3-1-1 Maidashi, Higashi-ku, Fukuoka 812-8582 Japan; Department of Orthopaedic Surgery, Kyoto University Graduate School of Medicine, 54 Kawahara-cho, Shogoin, Sakyo, Kyoto 606-8507 Japan

**Keywords:** Patellofemoral (PF), The tangent of the most distal part of femoral condyles, The tibial attachment of the patellar tendon, Medially prominent femoral component, Symmetric

## Abstract

**Background:**

The configuration of the distal surface of the femur would be more important in terms of the patellofemoral (PF) joint contact because the patella generally contacts with the distal surface of the femur in knee flexion. Some total knee arthroplasty (TKA) designs configurate medially prominent asymmetric femoral condyles. This difference in the design of distal femoral condyle may affect the PF joint congruity in knee flexion. Furthermore, some surgeons advocate a concept aligning the symmetric components parallel to the native joint inclination, not perpendicular to the mechanical axis. This concept would also make a difference on the PF joint congruity at the distal femur in knee flexion. However, no fundamental study has been reported on the PF congruity at the distal femur to discuss the theoretical priority of these concepts.

The current study investigated the angular relationship between the tibial attachment of the patellar tendon and the distal surface of the femur at 90° of flexion in normal knees.

**Methods:**

The open magnetic resonance images of 45 normal knees at 90° of flexion were used to measure the angles between the tibial attachment of the patellar tendon, the equatorial line of the patella, and the distal surface of femoral condyles.

**Results:**

The distal surface of femoral condyles was internally rotated relative to the tibial attachment of the patellar tendon and the equatorial line of the patella in all the knees (8.2° ± 3.5° and 5.8° ± 2.5°, respectively), not parallel.

**Conclusions:**

Distal femoral condyle is internally rotated to the patellar tendon at 90° of flexion in normal knees. When the symmetric femoral component is aligned perpendicular to the femoral mechanical axis, the patellar tendon would be possibly more twisted than the condition in normal knees, and the deviation of the PF contact force on the patellar component might be caused. The configuration and alignment of the distal condyle of the femoral component can affect the PF joint congruity in knee flexion. In this respect, our results provide important information in considering designs and alignment in the distal femur of TKA and the PF joint congruity in knee flexion.

## Background

Patellofemoral (PF) joint complication is one of the most frequent causes of revision total knee arthroplasty (TKA) [1,2]. With increasing numbers of patients who achieve deep knee flexion after TKA [3,4], more frequent PF problems can be expected because high contact forces are applied to the PF joint in knee flexion [5-7]. The patellar component generally contacts with the femoral component on its distal surface in knee flexion [8,9]; therefore, in terms of the PF joint contact forces, the configuration of the distal surface of the femoral component would be more important rather than the PF trochlear design which affects the PF forces in knee extension or slight flexed position [10]. The shape and size of the distal condyle of the femoral component can affect the contact configuration between the patellar component and the distal surface of the femoral component [10]. In the coronal view, the medial condyle is more distally prominent than the lateral condyle in normal knees [11] and some TKA designs take this anatomic feature into the design [12-14]. This difference in the design of distal femoral condyle may affect the congruity of patella to the distal femoral condyle while the knee is flexed. In addition, some surgeons advocate a concept aligning the symmetric components parallel to the native joint inclination, not in perpendicular to the mechanical axis [15-17]. This concept would also make a difference on the PF joint congruity at the distal femur in knee flexion. Knowledge of the normal knee anatomy regarding the congruity of patella to the distal femoral condyle is important to consider the influence of design and alignment of distal femoral condyle on the patellar kinematics in knee flexion. However, no fundamental study has been reported on the PF congruity at the distal femoral condyle.

The current study investigated the angular relationship between the tibial attachment of the patellar tendon and the distal surface of the femur at 90° of flexion in normal knees.

## Materials and methods

This study was approved by the institutional review board. The subjects were 22 Japanese and two Chinese volunteers (15 males and nine females; mean age, 31.6 years; range, 28–36 years) who had no knee symptoms, and bilateral knees of those subjects were examined. Three knees were excluded because one of those had a history of anterior cruciate ligament (ACL) operation, and the other two had a history of meniscus injury. Consequently, we examined 45 normal knees without history of injury. Their clinical status and magnetic resonance imaging (MRI) showed no abnormalities in their menisci, cartilages, or ligaments. They gave informed consent and agreed to participate in this study without payment.

**Figure 1:**
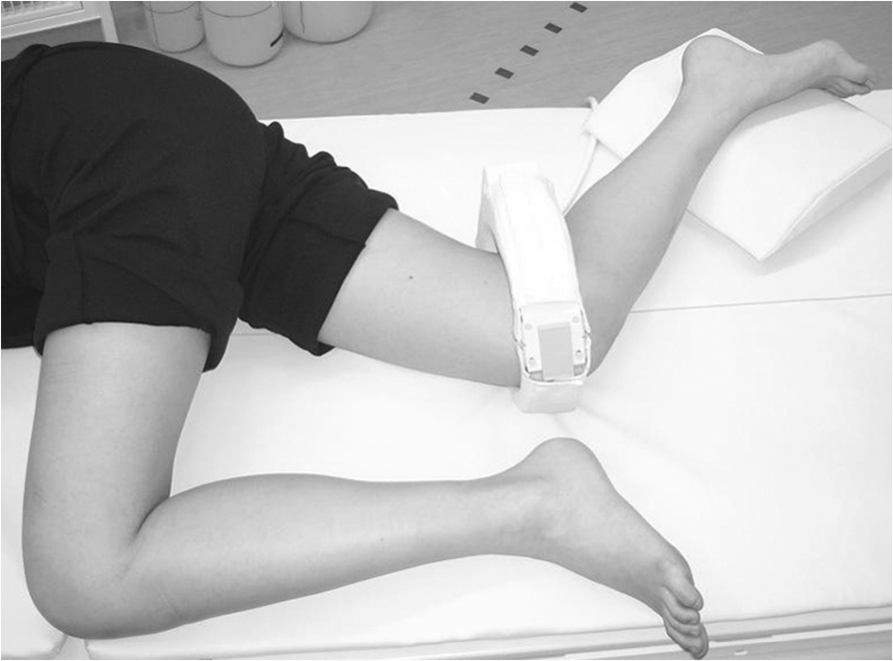
The knees were scanned at a knee flexion angle of 90°.

The MRI system used in this study was an open MRI at 0.4 T (APERTO; Hitachi Medical Corporation, Tokyo, Japan). The MRI was open in the horizontal direction with a 38-cm vertical gap. The subject was placed on the table and asked to lie on the side of the knee being examined. To stabilize the trunk and leg during the procedure, the contralateral hip and knee were flexed over and anterior to the knee under examination (Figure 1). The knee was flexed in 90°, and its position was chosen so that the subject felt a naturally flexed knee position without any feeling of internal or external rotation. The knee was scanned in the axial plane (TR/TE, 880 ms/19.0 ms; flip angle, 90°; field of volume, 200 mm; thickness, 2.0 mm). We took the MRI images as the DICOM data from the open MRI system server. The MRI data were modified to be perpendicular to the proximal tibial axis using a computer program, Real INTAGE V4.34 (CYBERNET SYSTEMS CO., LTD., Tokyo, Japan).

**Figure 2:**
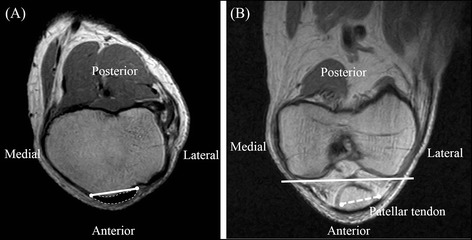
Measurement on MRI. **(A)** The medial and lateral borders of the patellar tendon at the tibial attachment level (white points) were identified, and the line between these borders was drawn (white line). **(B)** The most distal points of bilateral femoral condyles were identified, and the line between these points was drawn (white solid line). The line of the patellar tendon was projected (white broken line). The angle between these lines was measured.

First, the angle between the patellar tendon at the tibial attachment and the tangent of the most distal part of femoral condyles was measured. The patellar tendon at the tibial attachment level was identified, and the line between the medial and lateral border of the patellar tendon was drawn (Figure 2A). The most distal points of bilateral femoral condyles were identified, and the line between these points was drawn (Figure 2B). The line of the patellar tendon at the tibial attachment was projected onto this plane, and the angle between these lines was measured (Figure 2B).

**Figure 3:**
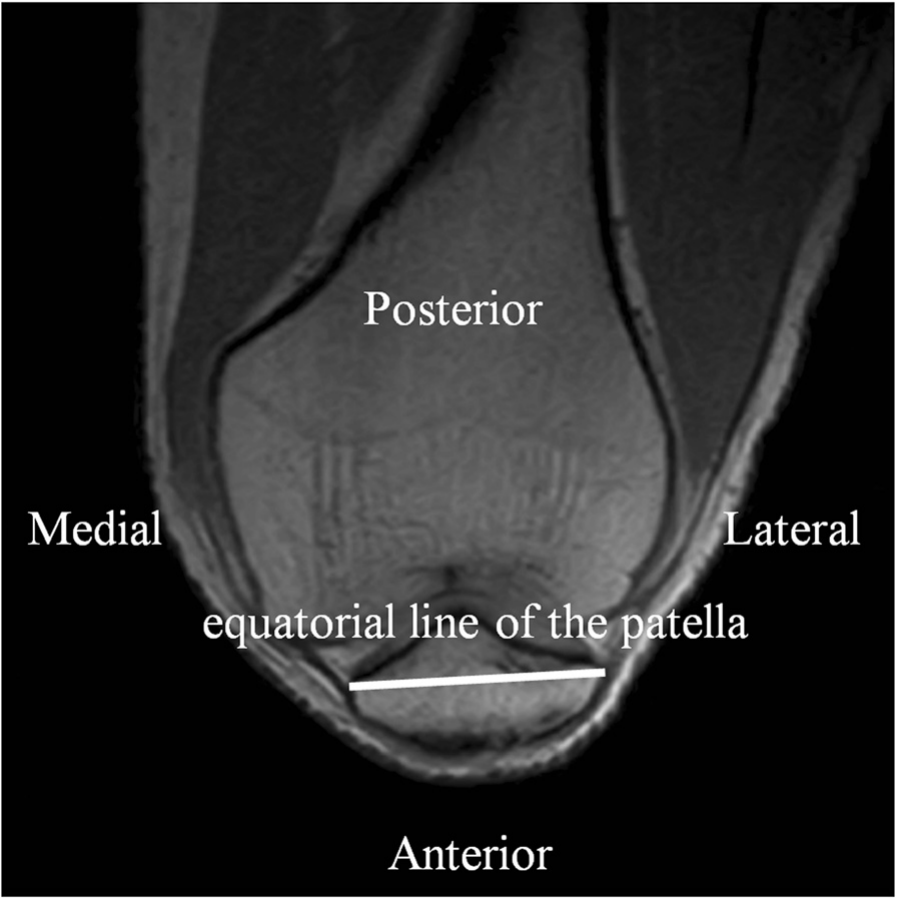
The line between the medial and lateral borders of the patella (the equatorial line of the patella, white solid line) was drawn.

Second, the angle between the tangent of the most distal part of femoral condyles and the equatorial line of the patella was measured. The plane in which the horizontal width of the patella was maximal was identified, and the line between the medial and lateral border of the patella in the plane was defined as the equatorial line of the patella (Figure 3) [18,19]. The angle between the tangent of the most distal part of femoral condyles and this line was measured.

To confirm the reproducibility of the knee position in MRI scanning, five knees randomly selected from the study group were scanned three times, and the angles between the patellar tendon at the tibial attachment and the most distal surface of the femoral condyles were each measured, respectively. A maximal difference between the angles of the same knee scans was less than 2.0°.

To evaluate the intraobserver and interobserver reproducibility, the measurement was performed three times by one examiner (SK) and once by two examiners (HN, SO) on the ten knees randomly selected from the study group. An intraclass correlation coefficient and an interclass correlation coefficient were calculated to test the reproducibility with an ANOVA under general linear model. The intraclass correlation coefficient among the three measurements conducted by the same observer (SK) was 0.92 for measurement of the angle between the patellar tendon and the tangent of the most distal part of femoral condyles and 0.83 for measurement of the angle between the tangent of the most distal part of femoral condyles and the equatorial line of the patella. The interclass correlation coefficient was calculated from the data of the measurements of two of the observers (HN, SO) and the average of the three measurements of the other observer (SK) and 0.87 for measurement of the angle between the patellar tendon and the tangent of the most distal part of femoral condyles and 0.81 for measurement of the angle between the tangent of the most distal part of femoral condyles and the equatorial line of the patella.

## Results

**Figure 4:**
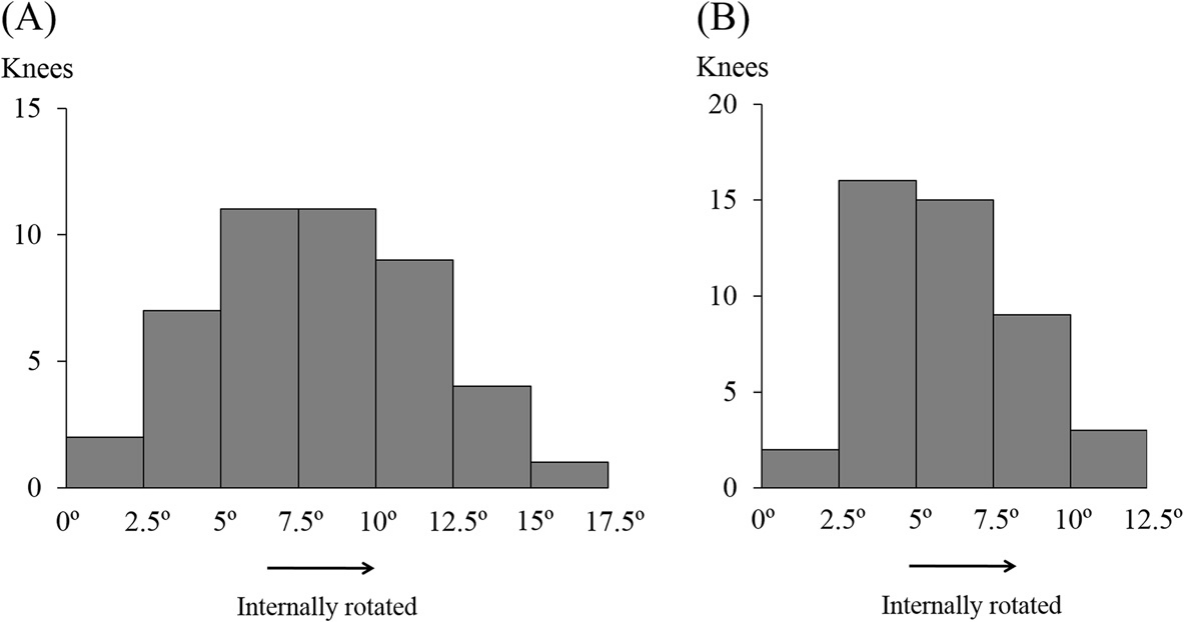
Distribution histograms show the amount of internal or external rotation of the tangent of the most distal part of femoral condyles. **(A)** Relative to the patellar tendon at the tibial attachment. **(B)** Relative to the equatorial line of the patella.

The tangent of the most distal part of femoral condyles was internally rotated relative to the patellar tendon at the tibial attachment in all the knees, averagely in 8.2° ± 3.5° (range, 1.8° to 17.3°, Figure 4A). There was no significant difference between gender (7.7° ± 2.9° in males and 9.7° ± 3.8° in females). The tangent of the most distal part of femoral condyles was also internally rotated relative to the equatorial line of the patella in all the knees, averagely in 5.8° ± 2.5° (range, 1.1° to 11.8°, Figure 4B). There was no significant difference between gender (5.3° ± 2.1° in males and 6.1° ± 3.0° in females). Angle differences between right and left knees were not significant in average in all parameters. However, five volunteers had angle differences more than 3° (8.5° in maximum) regarding the angle between the patellar tendon at the tibial attachment and the tangent of the most distal part of femoral condyles, and five volunteers had angle differences more than 3° (5.4° in maximum) regarding the angle between the tangent of the most distal part of femoral condyles and the equatorial line of the patella.

## Discussion

**Figure 5:**
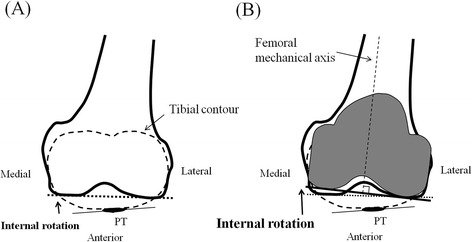
Schematic figures of the distal femur and the proximal tibia at a knee flexion angle of 90° seen from the ankle joint. **(A)** Preoperative condition. The tangent of the most distal part of femoral condyles (broken line) was internally rotated relative to the patellar tendon (solid line). **(B)** Postoperative condition with the symmetric femoral component. The tangent of the most distal part of the symmetric femoral component (bold solid line) would be more internally rotated relative to the patellar tendon (thin solid line).

High contact forces are applied to the PF joint in knee flexion [5-7]. In considering the PF joint congruity in knee flexion, the configuration of the distal surface of the femur would be more important rather than the PF trochlear configuration which affects the PF forces in knee extension or slight flexed position because the patella generally contacts with the femur on its distal surface [10]. In addition, the patella is connected to the tibial tuberosity by the patellar tendon; therefore, the positional relationship between the distal surface of the femur and the patellar tendon at the tibial attachment would be one of the important information in considering the PF joint congruity in knee flexion. This study shows that the tangent of the most distal part of femoral condyles was internally rotated relative to the patellar tendon at the tibial attachment and the equatorial line of the patella at 90-degree flexion in all the knees, not parallel (Figure 5A).

In TKA, rotational alignment of the femoral and tibial components, the configuration of the patellar component, and resurfacing the patella or not would be main factors affecting the PF joint congruity. However, in knees with correct rotational alignment, the shape and size of the distal condyle of the femoral component can affect the contact configuration between the patellar component and the distal surface of the femoral component [10]. In the coronal view, the medial condyle is more distally prominent than the lateral condyle in normal knees [11], and some TKA designs take this anatomic feature into the design [12-14]. Almost all of the effects of this asymmetric design have been described about the kinematics in the femorotibial joint, not in the PF joint in knee flexion. The difference between the distal surface of symmetric femoral component and medially prominent one may be one of factors affecting the PF joint congruity.

The tangent of the most distal part of femoral condyles was internally rotated relative to the patellar tendon and the equatorial line of the patella. When the symmetric femoral component is aligned perpendicular to the femoral mechanical axis, the patellar tendon would be possibly more twisted than the condition in normal knees, and the deviation of the PF contact force on the patellar component might be caused (Figure 5B). In addition, the resection of the patella would be generally done parallel to the equatorial line of the patella [18,19]. Therefore, when the symmetric femoral component is used, the deviation of the PF contact force on the patellar component might be caused for the same theory. Some surgeons advocate a concept aligning the symmetric components parallel to the native joint inclination, not perpendicular to the mechanical axis [15-17]. The finding obtained in this study would support this concept in the viewpoint of the PF configuration in deep knee flexion although aligning the femoral component parallel to the posterior condylar line in this concept could worsen the PF alignment in a low flexion angle. The theory remains a matter of speculation because rotational kinematics between femur and tibia in TKA might be different from that in normal knee. Nevertheless, a number of previous studies suggest that the degree of internal rotation of tibia in knee flexion in the implanted knee is less than that in normal knees [20-22]. Therefore, it is possible that the angulation between the distal femur and patella or patellar tendon in knee flexion could become greater than that in normal knee if the symmetrical femoral component is aligned to the mechanical axis. Furthermore, if the tibial component is aligned internally rotated, the angulation between the distal femur and patella or patellar tendon in knee flexion could become greater and the PF congruity would get worse [23]. These anatomical features in the congruity of patella and distal femur in the normal knee should be taken into account for the consideration of design in the distal femur of TKA and its effect on the PF alignment in knee flexion.

The current study has some limitations. First, this study showed the angular relationship only in the knee flexed in 90° because it would be simple to consider the effect of the configuration of the distal surface of the femur on the PF joint congruity in knee flexion. However, continuous analyses such as the fluoroscopic analysis and the computer simulation may lead to more minute evaluations for the PF joint congruity. Second, the study population was limited to Asian subjects. The data included in the current study may be typical for knees of Asian subjects, and there may be anatomic differences from the Caucasian population [24,25]. Furthermore, there should be morphological variations among the normal knees even in the same race. Third, bilateral knees were examined as two knees among 21 volunteers who have no history of knee injuries. Angle differences between right and left knees were not significant in average in all parameters though several volunteers had angle differences more than 3°. We cannot conclude only from these results whether it is appropriate to treat bilateral knees as two knees; however, it would be one of limitations of this study. Fourth, we defined the most distal points of bilateral femoral condyles in the same plane, where bilateral femoral condyles are most distally prominent, and bilateral edges of the equatorial line of the patella in the same plane, which has the maximal horizontal width of the patella. Strictly speaking, they might be in different planes and it might cause a slight difference in specific figures. However, in this study, we dealt in the geometric result that the tangent of the most distal part of femoral condyles was internally rotated relative to the patellar tendon at the tibial attachment level and the equatorial line of the patella in all the knees, not specific figures. Therefore, we believe the fundamental discussion would not change greatly. Fifth, our MRI was performed without weight-bearing conditions. Relative rotational position between the distal femur and the proximal tibia would be changed slightly by quadriceps contraction under weight-bearing conditions; however, it would vary according to an individual quadriceps contraction force. Moreover, it is very difficult to take MRI images during weight bearing. Though our results would not be applicable simply to weight-bearing conditions, we believe that they give a preliminary consideration for the PF joint congruity in knee flexion. Lastly, there could be a little variation in the knee position in MRI scanning, though its position was chosen so that the subject felt a naturally flexed knee position without any feeling of internal or external rotation. However, its variation would also be present in daily activities, and the knee position which the subject felt naturally would be the most basic position. In addition, our reproducibility trial of the knee position demonstrated acceptable results. Therefore, we believe that the results of this study are sufficiently reliable.

## Conclusions

The current study showed that the tangent of the most distal part of femoral condyles was internally rotated relative to the patellar tendon at the tibial attachment and the equatorial line of the patella in all the knees, not parallel. In TKA, the configuration and alignment of the distal condyle of the femoral component can affect the PF joint congruity in knee flexion. In this respect, our results provide important information in considering the PF joint congruity in knee flexion.

## Abbreviations

PF: Patellofemoral
TKA: Total knee arthroplasty
MRI: Magnetic resonance imaging
